# The association of language barrier and depression among newly admitted US nursing home residents

**DOI:** 10.1093/geroni/igaf122.2717

**Published:** 2025-12-31

**Authors:** Natalia Nielsen, Maira Castañeda-Avila, David Dosa, Shao-Hsien Liu, William Jesdale, Yiyang Yuan, Jonggyu Baek, Kate Lapane

**Affiliations:** University of Massachusetts Chan Medical School, Worcester, Massachusetts, United States; University of Massachusetts Chan Medical School, Worcester, Massachusetts, United States; University of Massachusetts Chan Medical School, Worcester, Massachusetts, United States; University of Massachusetts Chan Medical School, Worcester, Massachusetts, United States; University of Massachusetts Chan Medical School, Worcester, Massachusetts, United States; University of Massachusetts Chan Medical School, Worcester, Massachusetts, United States; University of Massachusetts Chan Medical School, Worcester, Massachusetts, United States; University of Massachusetts Chan Medical School, Worcester, Massachusetts, United States

## Abstract

Language barriers result in negative outcomes for nursing home residents of diverse linguistic backgrounds. Yet, the relationship between language barriers and resident depression symptoms or clinical diagnosis is understudied. Coming decades will likely see a rise in older Latino adult nursing home admissions in the US. We compared the prevalence of moderate/severe depressive symptoms and clinical diagnosis of depression among English- and Spanish-speaking Latinos (L-E and L-S, respectively) and non-Latino English-speaking White residents (NLW-E). Newly admitted Latino and non-Latino White nursing home residents aged ≥50 years were included (n = 889,205). Generalized estimating equation models were used to estimate adjusted prevalence ratios (aPRs) and 95% confidence intervals (CI) for depression outcomes across racial/ethnic and language groups. Our sample was mostly White, female and ≥75 years. Of Latinos (n = 76,749), 25.8% indicated the need for a Spanish-speaking interpreter. The prevalence of moderate/severe depression was highest in Spanish-speaking Latino residents (NLW-E: 15.6%; L-E: 15.9%; L-S: 17.5%). After adjusting for covariates and accounting for facility level clustering, Latinos had lower prevalence of clinically diagnosed depression compared to non-Latino Whites (Latino vs. non-Latino White aPR: 0.93; 95% CI: 0.92-0.95) and Spanish-speaking Latinos had lower prevalence of clinically diagnosed depression compared to English-speaking Latinos (L-S vs L-E aPR: 0.95; 95% CI: 0.93-0.98). Similar results were found for moderate/severe depression (Latino vs. non-Latino White aPR: 0.93; 95% CI: 0.91-0.95; L-S vs L-E aPR: 0.95; 95% CI: 0.91-0.99). Future research to better understand the relationship between language barriers and depression in this growing population within US nursing homes is warranted.

